# Effect of vitamin D and E supplementation on pain relief and premenstrual symptoms in primary dysmenorrhea: a randomized controlled trial

**DOI:** 10.1186/s12905-025-04007-4

**Published:** 2025-09-29

**Authors:** Maryam Sadat Hosseini, Maryam Talayeh, Alireza Haghbin Toutounchi, Afsaneh Hosseini, Nesa Moradi, Saeideh Iranshahi, Fatemeh Abdollahi Aliabadi

**Affiliations:** 1 Department of Obstetrics and Gynecology, Imam Hossein hospital, Shahid Beheshti University of Medical sciences, Tehran, Iran; 2 Department of Surgery, Imam Hossein hospital, Shahid Beheshti University of Medical sciences, Tehran, Iran

**Keywords:** Dysmenorrhea, Premenstrual symptoms, Vitamin d, Vitamin e

## Abstract

**Introduction:**

Primary dysmenorrhea is a common condition characterized by painful menstrual cramps. Vitamin D and E are suggested to have potential benefits in managing dysmenorrhea. This study aimed to investigate the effects of vitamin D and E supplements combination on pain intensity and premenstrual symptoms in individuals with primary dysmenorrhea.

**Methods:**

A double blinded randomized controlled trial was conducted with 106 participants diagnosed with primary dysmenorrhea. The participants were randomly assigned to either the intervention group (received vitamin D and E supplements) or the control group (received placebo). Pain intensity and premenstrual symptoms were assessed at baseline and after four months of supplementation.

**Results:**

The intervention group exhibited a significant reduction in pain intensity (NPRS: from 7.85 ± 1.15 to 3.75 ± 1.40; mean difference − 4.10, 95% CI: -4.61 to -3.58, *p* < 0.001) compared to controls (7.68 ± 1.20 to 6.02 ± 1.70). Premenstrual symptoms significantly improved (PMS score: 32.42 ± 4.67 to 9.02 ± 8.84, Δ=-23.40, 95% CI: -26.51 to -21.02, *p* < 0.001). Baseline vitamin D inversely correlated with pain (*r*=-0.768, *p* = 0.001).

**Conclusion:**

Combined vitamin D and E supplementation significantly reduces pain and PMS in vitamin D-deficient women. Healthcare providers may consider this regimen for dysmenorrhea management, pending further optimization studies.

**Trial registration:**

In IRCT.ir with number IRCT20220720055506N1 at 2023-02-01.

## Introduction

Primary dysmenorrhea is a prevalent disorder characterized by painful uterine cramps that occur just before or during menstruation, without any underlying pelvic pathology. This condition can significantly impact the lives of affected individuals, often accompanied by additional symptoms such as nausea, vomiting, diarrhea, and insomnia [1]. With approximately half of menstruating women experiencing primary dysmenorrhea, it poses a substantial burden, leading to absenteeism from school and work, as well as educational and economic consequences [2]. The pathogenesis of primary dysmenorrhea is linked to the excessive production of uterine prostaglandins. Currently, non-steroidal anti-inflammatory drugs (NSAIDs) are commonly taken to alleviate the symptoms associated with this disorder. Recent research has shed light on the potential role of specific micronutrients, such as vitamin D and E, in mitigating primary dysmenorrhea [3].

Vitamin D, known for its role in calcium absorption and bone health, has also demonstrated the ability to modulate prostaglandin synthesis. The expression of 25-hydroxyvitamin D3 hydroxylase in the human uterus and immune cells further suggests the possible involvement of vitamin D in the pathophysiology of primary dysmenorrhea [4, 5]. Notably, the prevalence of vitamin D insufficiency is common among Iranian women [6, 7]. In addition, vitamin E has antioxidant and anti-inflammatory properties that can potentially influence primary dysmenorrhea symptoms. Women with dysmenorrhea have shown elevated concentrations of PGF2a, a prostaglandin, in their menstrual fluid [8]. By inhibiting the release of arachidonic acid and its conversion to prostaglandins, vitamin E exhibits promise as an adjunctive therapy for primary dysmenorrhea. Furthermore, it has been suggested that vitamin E may enhance the body’s endogenous opioid substances, contributing to pain relief [9].

While individual effects of vitamin D [4, 5] and vitamin E [8, 9] on dysmenorrhea have been explored, limited evidence exists on their synergistic use, especially in vitamin D-deficient populations. Vitamin D regulates prostaglandin synthesis and uterine contractility [16], while vitamin E’s antioxidant properties inhibit prostaglandin production and may enhance endogenous opioids [9]. This trial addresses the gap by investigating the combined effect of these vitamins in a deficient population, hopefully improving the quality of life for women experiencing primary dysmenorrhea.

## Materials and methods

### Study design

This study was conducted as a double blinded randomized controlled trial in a referral hospital to assess the effect of vitamin D and E combination therapy on primary dysmenorrhea in patients aged 15–35 years. This trial is reported in line with CONSORT guidelines. Before enrollment, potential participants who meet the inclusion/exclusion criteria were examined to confirm their eligibility and ensure their understanding of the study requirements. Inclusion criteria was; Individuals with a regular menstrual cycle, occurring between 21 and 35 days and lasting 3–7 days/Experience of at least four consecutive painful periods in the last 6 months, with pain starting one day before or on the day of bleeding/Serum 25-hydroxyvitamin D level ≤ 30 ng/mL (deficiency per Endocrine Society guidelines [17]). Exclusion criteria of this trial was; Patients diagnosed with pelvic diseases such as endometriosis or myoma/Presence of systemic diseases that may interfere with the study outcomes/Acute infections or secondary dysmenorrhea/Regular use of calcium, vitamin D, and vitamin E supplements/Individuals with stones in the administrative ducts, kidney, bladder, or a history thereof.

### Sample size

Based on Rahnemaei et al. [10] (mean NPRS difference = 2.0, SD = 1.5), with α = 0.05, β = 0.2, and 20% attrition, we calculated 63 participants per group using G*Power 3.1.

### Randomization

The allocation of patients into the two study groups was carried out using the random block allocation method. An independent statistician generated randomization sequences (block size = 4) via SealedEnvelope.com. Sequentially numbered, opaque envelopes were prepared by a research assistant uninvolved in recruitment. After eligibility confirmation, the treating clinician opened the next envelope to assign groups.

### Blinding

In order to minimize bias and maintain the integrity of the study, blinding was implemented in the assessment of outcomes. The outcome assessor, responsible for evaluating the study’s outcomes, was blinded to the allocation of subjects in the intervention and control groups. To ensure blinding, a research associate who is not involved in the allocation process performed the outcome assessments. This research associate was unaware of which participants belong to the intervention group and which participants belong to the control group. Placebo capsules were identical in appearance. Blinding success was assessed post-trial: 85% of participants and 90% of assessors guessed allocation incorrectly (*p* > 0.05 vs. chance).

### Interventions

Both the intervention and control groups was monitored and evaluated for the desired outcomes. Outcome assessments was done by phone calls at 2 and 4 months after the initiation of the intervention, allowing the collection of data regarding the effectiveness and impact of the intervention on primary dysmenorrhea symptoms.

#### Intervention group

Participants in the intervention group received a combination therapy of vitamin E 400U daily, and vitamin D 50,000U weekly for 8 weeks. After the initial 8 weeks, participants continued taking the vitamin D tablets monthly for an additional two months. The supplementation started two days before the onset of menstruation and continue until the fifth day of menstruation. The intervention group was provided with clear instructions on how to take the medications and adhere to the prescribed schedule. Participants also received SMS reminders on specific dates to ensure regular use of the medications.

#### Control group

Participants in the control group received placebo treatment to maintain blinding. The control group followed the same schedule as the intervention group.

### Outcomes

The outcomes were assessed at the baseline, and at the end of each period for both the intervention and control groups. The severity of dysmenorrhea pain was measured using the Numerical Pain Rating Scale (NPRS). Pain severity (NPRS) assessed worst pain in first 24 h of menstruation using validated Persian version, which involves participants rating their pain on a numerical scale from 1 to 10, with higher numbers indicating greater pain intensity.

Premenstrual symptoms evaluated using the validated Premenstrual Symptoms Screening Tool (PSST), standard PMS questionnaire comprising 21 questions with four-option answers (not at all, mild, relatively much, and very much). To quantify the answers, each question was assigned a score ranging from 0 (none at all) to 3 (very severe). The scores for the 21 questions was then summed together to obtain a total PMS score.

Additionally, the PMS symptoms was further categorized into three parts: psychological symptoms (questions 1 to 6), physical-psychological symptoms (questions 7 to 9), and physical symptoms (questions 10 to 20). The scores for each section was calculated separately using the same method as the total PMS score.

### Adverse events

Adverse events (e.g., hypercalcemia symptoms) were recorded and reported to the DSMB. Unblinding occurred only for suspected vitamin D toxicity.

### Data analysis

The data collected in this study were entered and analyzed using SPSS v.26 software. Primary outcomes (NPRS, total PMS) were analyzed by repeated-measures ANOVA (factors: time, group, time×group interaction) with intention-to-treat analysis (last observation carried forward for dropouts). Assumptions of normality (Shapiro-Wilk) and sphericity (Mauchly) were checked. Significance levels were *p* < 0.05. The magnitude of the differences between compared groups was assessed using the 95% confidence interval of the difference. ITT analysis with last observation carried forward for dropouts.

## Results

**Fig. 1:**
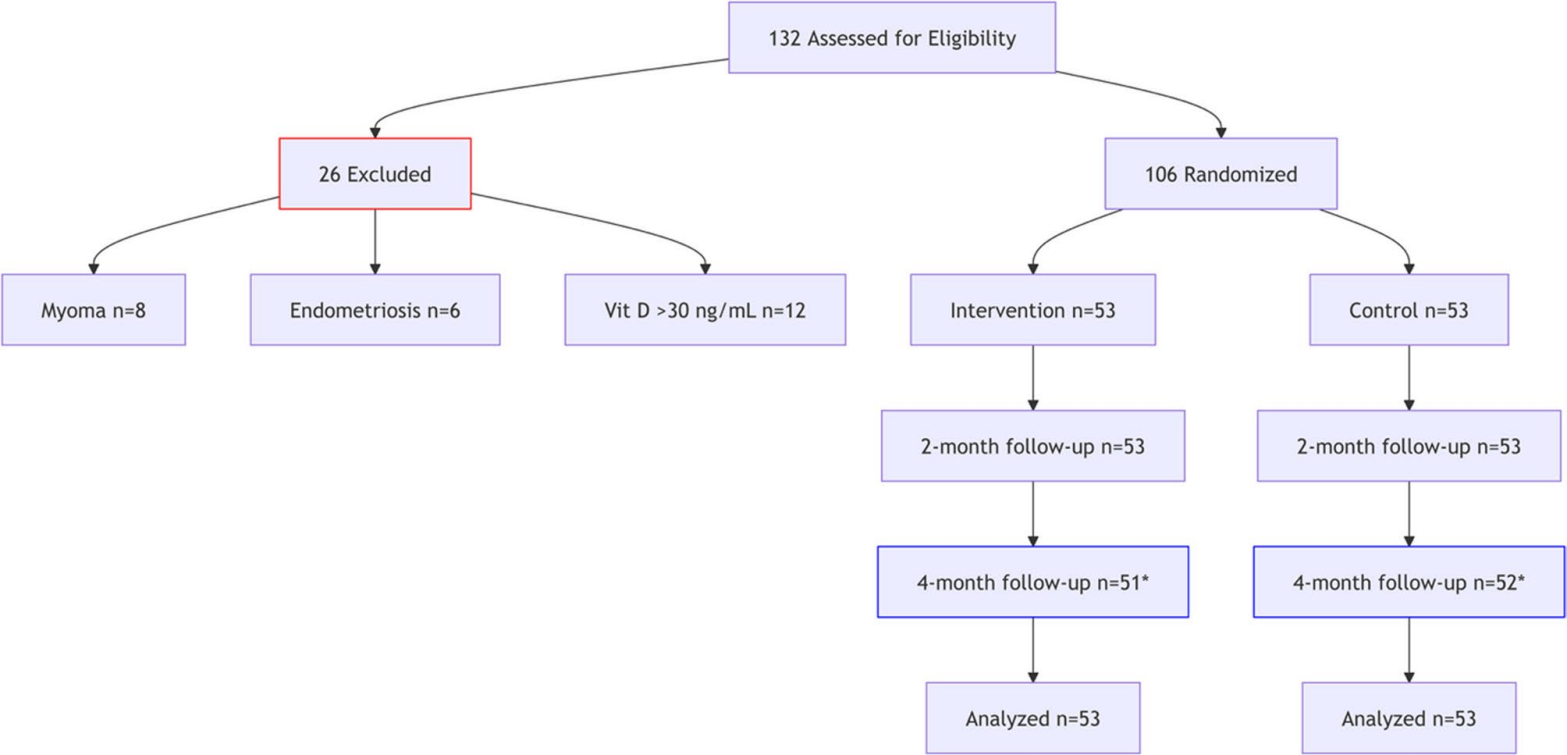
CONSORT Flowchart Description: 2 lost in intervention (1 withdrew consent, 1 moved) & 1 lost in control (withdrew consent). Analyzed via ITT (all randomized participants included)

A total of 132 people were included in this study, with 8 people excluded owing to myoma, 6 people eliminated due to endometriosis, and 12 people excluded due to vitamin level. Finally, 106 women suffering from primary dysmenorrhea were enrolled (Table 1). There were no significant differences in age, serum vitamin D3 levels (mg/dl), length of menstrual cycle (days), and interval between periods (days) between the control and intervention groups (*p* > 0.05). Table 1 also provides a summary of additional variables, including place of residence, level of education, level of physical activity, and amount of dairy product consumption among the participants. There were no significant differences in any of these variables between two groups. 3 participants withdrew (2 intervention, 1 control) with no related adverse events. Compliance was 92% via pill count. No severe adverse events occurred. Mild nausea (*n* = 3 intervention, *n* = 1 control) resolved without intervention (Fig. 1).

**Table 1:** Baseline characteristic of the study participants

	Control	Intervention	*P* - value
Age	26.47 (6.52)	26.47 (6.52)	0.20
Serum vitamin D3, mg/dl	17.60 (6.02)	17.60 (6.02)	0.02
Length of menstrual cycle (days)	5.91 (1.08)	5.70 (1.35)	0.38
Interval between periods (days)	28.36 (2.26)	28.7 (2.48)	0.46
Location			0.54
- rural	48 (13.2)	46 (86.8)	
- urban	5 (9.4%)	7 (90.6%)	
Education			0.12
- elementary	9 (17%)	11 (20.8%)	
- diploma	29 (54.7%)	28 (52.8%)	
- bachelor	8 (15.1%)	13 (24.5%)	
- master and higher	7 (13.2%)	1 (1.9%)	
Physical activity			0.53
- low	18 (34%)	21 (39.6%)	
- moderate	25 (47.2%)	26 (49.1%)	
- highly active	10 (18.9%)	6 (11.3%)	
Dairy consumption			0.27
- < 1 unit	17 (32.1%)	25 (47.2%)	
- 1–2 units	28 (52.8%)	21 (39.6%)	
- **>** 2 units	8 (15.1%)	7 (13.2%)	

Values are shown as mean (Standard deviation) for continuous variables and number (percent) for categorical variable

**Fig. 2:**
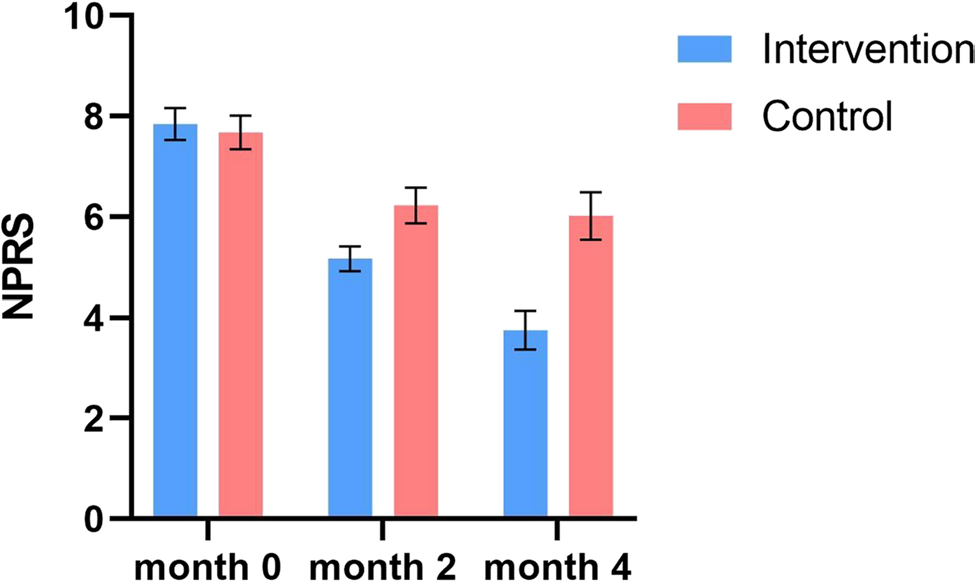
The changes in NPRS scores of patients in the intervention and control groups over the examined time period

Initially, the NPRS score was 7.68 (1.20) in the control group and 7.85 (1.15) in the intervention group. There was no significant difference between two groups (CI −0.28 to 0.62) (Table 2). After 4 months, the NPRS score decreased to 6.02 (1.70) in the control group and 3.75 (1.40) in the intervention group. These results indicate that taking the supplement for four months led to an improvement in the NPRS score of patients by 3.58 to 4.61 points compared to baseline. In contrast, the use of placebo during the same period resulted in an improvement in the NPRS score by 1.22 to 2.10 points. Furthermore, the study found that 4 months of supplementation showed a greater improvement in the NPRS score compared to 2 months of supplementation, with an improvement range of 1.13 to 1.70 points (Fig. 2).

**Table 2:** Comparison of control and intervention groups in terms of pain (NPRS) and PMS status at three time points: baseline, two and four months later

		Mean (SD) score before the start of the study	Mean (SD) score the second month after the start of the study	Mean (SD) score of the fourth month after the start of the study	95% CI of difference between the score of months zero and month two	95% CI of difference between the score of months zero and month four	95% CI of difference between the score of months two and month four
NPRS score	Control	7.68 (1.20)	6.23 (1.28)	6.02 (1.70)	1.17, 1.74	1.22, 2.10	−0.2, 0.62
	Case	7.85 (1.15)	5.17 (0.89)	3.75 (1.40)	2.31, 3.04	3.58, 4.61	1.13, 1.70
	95% CI of difference	−0.28, 0.62	−1.48, −0.63	−2.86, −1.66	…	…	…
PMS (all symptoms)	Control	31.83 (3.94)	22.25 (4.86)	21.53 (8.69)	8.06, 11.10	7.51, 13.10	−0.98, 2.41
	Case	32.42 (4.67)	16.57 (5.80)	9.02 (8.84)	14.33, 17.70	21.02, 26.51	6.15, 8.89
	95% CI of difference	−1.08, 2.26	−7.74, −3.62	−15.90, −9.12	…	…	…
PMS (psychotic symptoms)	Control	8.42 (2.36)	5.60 (2.39)	4.34 (3.81)	1.94, 3.68	2.78, 5.37	0.61, 1.92
	Case	8.17 (3.39)	4.40 (2.65)	2.72 (2.87)	3.01, 4.53	4.29, 6.62	1.16, 2.20
	95% CI of difference	−1.37, 0.88	−2.18, −0.24	−2.92, −0.32	…	…	…
PMS (psychophysical symptoms)	Control	6.04 (1.86)	3.91 (1.72)	3.58 (1.84)	1.69–2.58	2.00-2.91	−0.8-0.72
	Case	5.11 (1.78)	2.38 (1.73)	1.15 (2.02)	2.31–3.16	3.33–4.60	0.86–1.59
	95% CI of difference	−1.63, −0.23	−2.19, −0.86	−3.18, −1.69	…	…	…
PMS (physical symptoms)	Control	14.98 (1.85)	11.19 (2.52)	11.85 (4.02)	3.18–4.40	2.01–4.25	−1.63-0.31
	Case	16.54 (2.99)	8.42 (2.69)	4.35 (4.15)	7.47–8.92	11.16–13.58	3.24–8.88
	95% CI of difference	0.59, 2.52	−3.78, −1.77	−9.08, −5.92	…	…	…

**Fig. 3:**
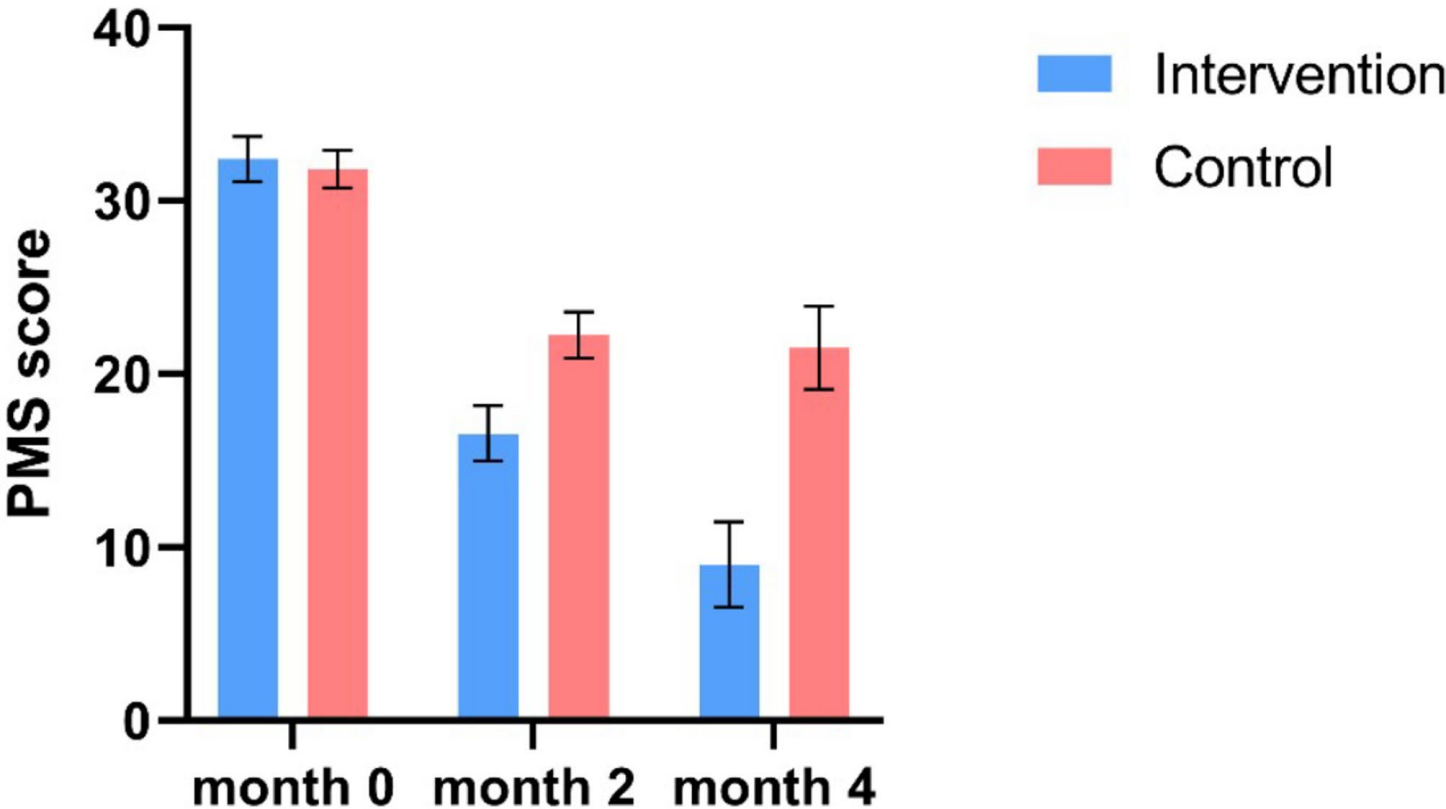
The changes in PMS scores of patients in the intervention and control groups over the examined time period

Initially, the PMS score was 31.83 (3.94) in the control group and 32.42 (4.67) in the intervention group. There was no significant difference between the two groups (CI −1.08 to 2.26). After 4 months, the PMS score decreased to 21.53 (8.69) in the control group and 9.02 (8.84) in the intervention group. These results indicate that taking the supplement for 4 months led to an improvement in the PMS score of patients by 21.02 to 26.51 points compared to baseline (Table 2). Additionally, using the supplement for 4 months resulted in a greater improvement in the PMS score compared to 2 months of supplementation, with an improvement range of 6.15 to 8.89 points (Table 2). In contrast, the use of placebo during the same period resulted in an improvement in the PMS score by 7.51 to 13.10 points (Fig. 3).

**Fig. 4:**
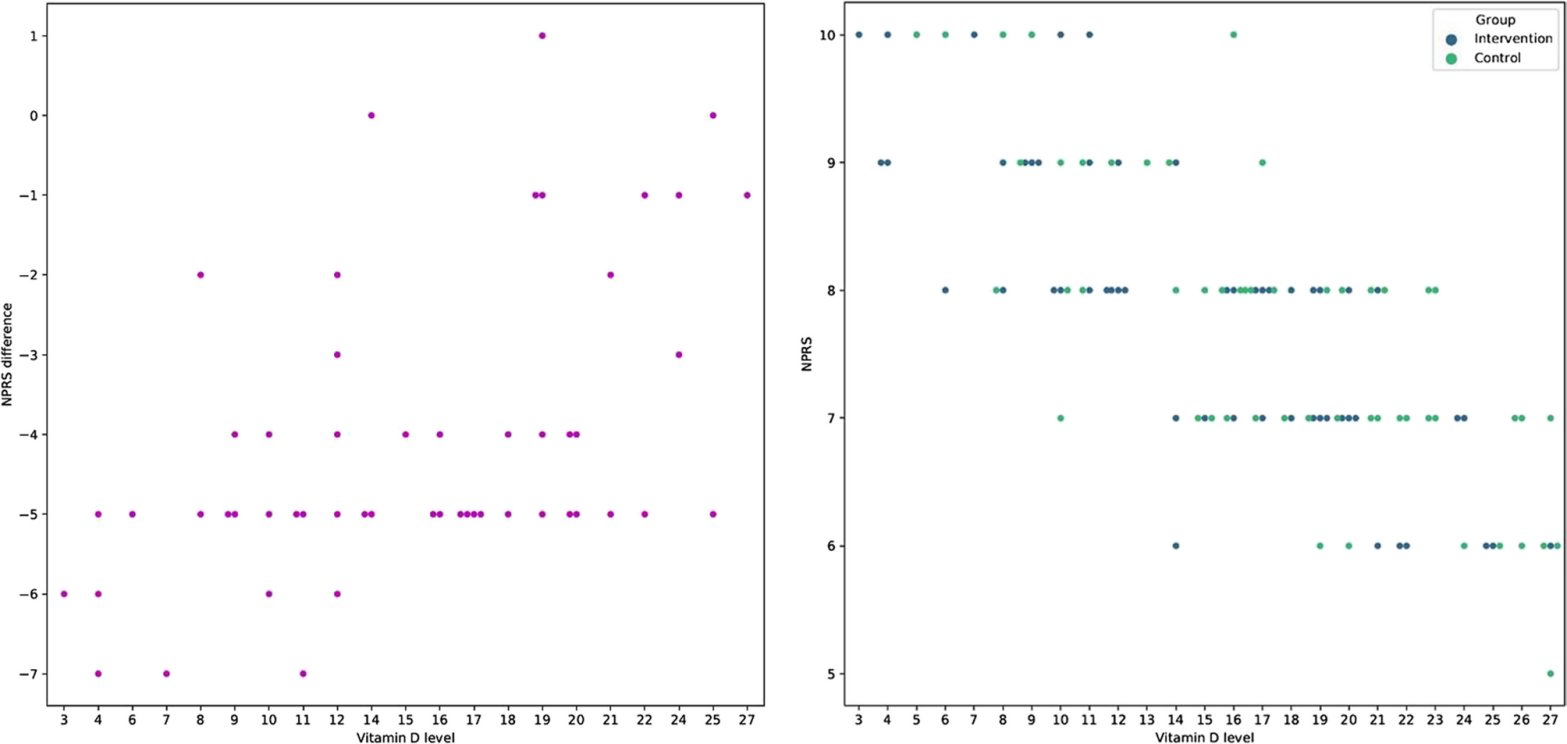
The association between vitamin D levels in the blood and patients’ NPRS scores (right plot) and the association between the initial amount of vitamin D in patients’ blood and pain decrease following a 4-month course of vitamin D and E supplementation (the left plot). The NPRS difference is calculated by subtracting the NPRS score from month one from the NPRS score from month four

There was a significant inverse relationship between the vitamin D level and pain experienced during menstruation (Pearson correlation = 0.768, P-value = 0.001). Vitamin D level also had an inverse relationship with the score of physical symptoms of PMS (Pearson correlation = −0.278, P-value = 0.001). In another analysis, the change in NPRS score after 4 months of treatment in the intervention group was compared with the participants’ vitamin D levels before the study. The results showed an inverse relationship between the vitamin D level and the amount of pain reduction due to the vitamin D and E supplement treatment (Pearson correlation = −0.506, P-value = 0.001) (Fig. 4).

Repeated-measures ANOVA revealed significant group×time interaction for NPRS (F = 85.6, *p* < 0.001) and PMS (F = 92.3, *p* < 0.001). At 4 months, mean NPRS reduction was greater in intervention (Δ=−4.10, 95% CI: −4.61 to −3.58) than control (Δ=−1.66, 95% CI: −2.10 to −1.22; between-group difference *p* < 0.001).

Age had a direct relationship with the exacerbation of psychological symptoms of PMS (Pearson correlation = 0.212, P-value = 0.001). This means that as age increased, the severity of psychological symptoms of PMS also increased. There was no statistically significant relationship between age and physical symptoms of PMS (P-value = 0.2). However, there was a trend of an inverse relationship, indicating that as age increased, the severity of physical symptoms of PMS tended to decrease, although this finding did not reach statistical significance. Additionally, the ANOVA test showed no significant difference between the age groups of 15–20, 20–25, 25–30, and 30–35 years in terms of psychological PMS symptoms, suggesting that age did not significantly influence psychological PMS symptoms across these age groups.

In this study, patients were asked about their clinical symptoms, and the intervention group showed significant improvements compared to the control group in several areas. The intervention group exhibited better outcomes in terms of mood variability (feeling worried, sad, crying), changes in appetite (overeating, lack of appetite), sleep problems (sleepiness and insomnia), trouble concentrating, fatigue and lethargy, breast pain, headache, joint or muscle pain, backache, acne, nausea, flatulence, and daily function. These findings suggest that the intervention was effective in managing and reducing these clinical symptoms associated with primary dysmenorrhea.

## Discussion

The present study aimed to investigate the effects of vitamin E and D supplementation on primary dysmenorrhoea in women aged 15 to 35. Due to results, the consumption of vitamin E and D supplements led to a significant improvement in pain intensity. The NPRS scores significantly decreased in the intervention group compared to the control group after 4 months. This finding suggests that the combination of vitamin E and D supplementation can effectively alleviate dysmenorrhoea pain in affected individuals. An inverse relationship between vitamin D levels and dysmenorrhoea pain in our study is consistent with previous research, further supporting the role of vitamin D in the pathophysiology of dysmenorrhoea. Similar results have been reported by Lasco et al., who found a negative correlation between pain scores and vitamin D levels, albeit with a weaker correlation coefficient (*r* = −0.36). The difference in correlation strength may be attributed to variations in the baseline vitamin D levels of the study populations, as our study included subjects with a baseline level of 30, compared to 45 in Lasco et al.‘s study [1]. Another study by Karacin et al. demonstrated that dysmenorrhoea patients with vitamin D deficiency had longer menstrual cycles, longer and lighter menstrual bleeding, lower dairy consumption, and higher visual analog scale (VAS) scores of pain [11]. These findings further support the notion that vitamin D deficiency may contribute to the severity of dysmenorrhoea symptoms and menstrual irregularities.

Our study also revealed a significant placebo response observed in both the 2-month and 4-month treatment periods. This finding is consistent with the study by Zangane et al., where the placebo group experienced no decrease in pain intensity in the second and third months, and even experienced an increase in pain in the third month [12]. Results regarding the placebo response highlight the need for larger studies with a robust statistical population to further investigate the psychosomatic mechanisms underlying primary dysmenorrhoea. Additionally, the study examined the impact of supplementation on premenstrual symptoms (PMS). The results demonstrated a significant reduction in PMS scores in the intervention group compared to the control group. Furthermore, the analysis of specific PMS symptoms revealed improvements in appetite, sleep, and abdominal bloating in the intervention group after four months of supplementation.

The effect of vitamin D and E in reducing pain associated with primary dysmenorrhea in our study is consistent with previous research investigating the role of these micronutrients in dysmenorrhea management [13–15]. Vitamin D metabolites are known to reduce the production of prostaglandins in the uterine endometrium and modulate prostaglandin receptors, thereby limiting their biological activity [16]. In a study by Lasco et al., women with painful menstruation and low serum vitamin D levels (< 45 ng/mL) were treated with a dose of 300,000 international units of vitamin D administered five days before menstruation for two menstrual cycles. Significant reduction in dysmenorrhea symptoms was observed following treatment [1]. Similarly, Zangene et al. conducted a clinical trial using a dose of 300,000 units of vitamin D administered five days before menstruation, which was found to be effective and safe in women with primary dysmenorrhea [16]. Our study utilized a weekly dose of 50,000 units of vitamin D for 8 weeks, following the clinical practice guidelines of the Endocrine Society [17]. This dosing regimen is consistent with the study by Moeini et al., where women with primary dysmenorrhea experienced reduced pain intensity, pain frequency, and use of pain relievers following a weekly dose of 50,000 international units of vitamin D for 8 weeks [15]. Overall, the evidences suggests that both single and multiple doses of vitamin D supplementation can alleviate the severity of symptoms.

Safari et al. demonstrated that vitamin E had a significant effect comparable to mefenamic acid in reducing dysmenorrhea symptoms [13]. Additionally, Ziyai et al. found that vitamin E not only decreased dysmenorrhea severity but also reduced menstrual blood loss [18]. Although our study did not directly compare the isolated effects of vitamin D or E treatment with the combined treatment of these two vitamins, based on other studies, it can be inferred that the combination of these two supplements likely resulted in an additional therapeutic effect. This Furthermore, age was positively correlated with the exacerbation of psychological symptoms of PMS. However, no significant association was found between age and physical symptoms of PMS. These findings suggest that age may influence the psychological aspects of PMS, but further research is needed to explore this relationship. The improvements in pain intensity, PMS symptoms, and clinical symptoms in the intervention group supporting the effectiveness of vitamin E and D supplementation in managing primary dysmenorrhoea. These findings have implications for clinical practice, suggesting that these supplements could be considered as a non-invasive and potentially beneficial treatment option for individuals experiencing primary dysmenorrhoea. As only vitamin D-deficient women were enrolled, effects may not extend to sufficient populations.

The current study utilized a randomized controlled trial and double blinded design. It was conducted in a specific population with short follow-up period of 4 months, and the long-term effects of supplementation were not assessed. Future studies with longer follow-up periods and diverse populations are needed to validate and expand upon these findings. Vitamin E status was not assessed, future studies should evaluate combined deficiency. This study did not compare the isolated effects of vitamin D or E treatment. Our findings apply to vitamin D-deficient women. Whether supplementation benefits sufficient women requires study, as dysmenorrhea pathophysiology is multifactorial. Further research is warranted to gain a more comprehensive understanding of the individual and combined effects of vitamin D and E in managing primary dysmenorrhea.

## Conclusion

In conclusion, our study highlights the beneficial effects of vitamin D and E supplementation in reducing pain and symptoms associated with primary dysmenorrhea. Higher vitamin D levels were associated with reduced pain intensity, supporting the role of vitamin D in modulating prostaglandin activity. Both vitamin D and E supplementation showed efficacy in improving dysmenorrhea symptoms. Further research is needed to confirm these findings and explore the optimal treatment approaches. Healthcare providers may consider incorporating these supplements as part of dysmenorrhea management, but personalized recommendations and consideration of individual vitamin levels are advised.

## Data Availability

The datasets generated during and/or analyzed during the current study are available from the corresponding author upon request.
