# Correction: Effect of vitamin D and E supplementation on pain relief and premenstrual symptoms in primary dysmenorrhea: a randomized controlled trial

**DOI:** 10.1186/s12905-025-04094-3

**Published:** 2025-10-24

**Authors:** Maryam Sadat Hosseini, Maryam Talayeh, Alireza Haghbin Toutounchi, Afsaneh Hosseini, Nesa Moradi, Saeideh Iranshahi, Fatemeh Abdollahi Aliabadi

**Affiliations:** 1 Department of Obstetrics and Gynecology, Imam Hossein hospital, Shahid Beheshti University of Medical sciences, Tehran, Iran; 2 Department of Surgery, Imam Hossein hospital, Shahid Beheshti University of Medical sciences, Tehran, Iran

**Correction: BMC Women’s Health 25, 455 (2025)**

**https://doi.org/10.1186/s12905-025-04007-4**

In this article [1], Figs. 1, 2, 3 and 4 were wrongly numbered. Figure 1 should have been Fig. 4; Fig. 2 should have been Figs. 1 and 3 should have been Figs. 2 and 4 should have been Fig. 3. The correct version of the Figs. 1, 2, 3 and 4 with approiate caption are shown below. The original article has been corrected.

**Fig. 1:**
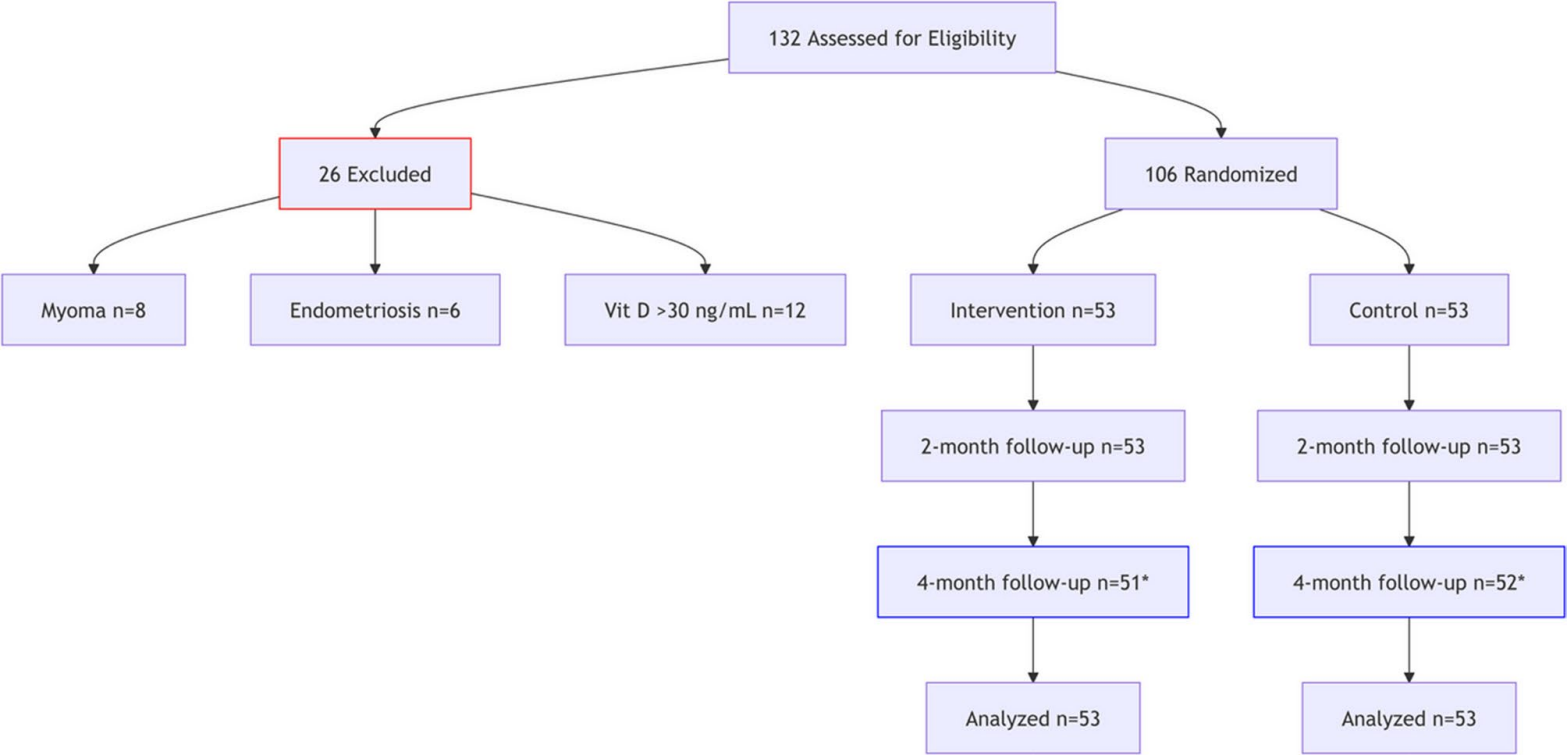
CONSORT Flowchart Description: 2 lost in intervention (1 withdrew consent, 1 moved)& 1 lost in control (withdrew consent). Analyzed via ITT (all randomized participants included)

**Fig. 2:**
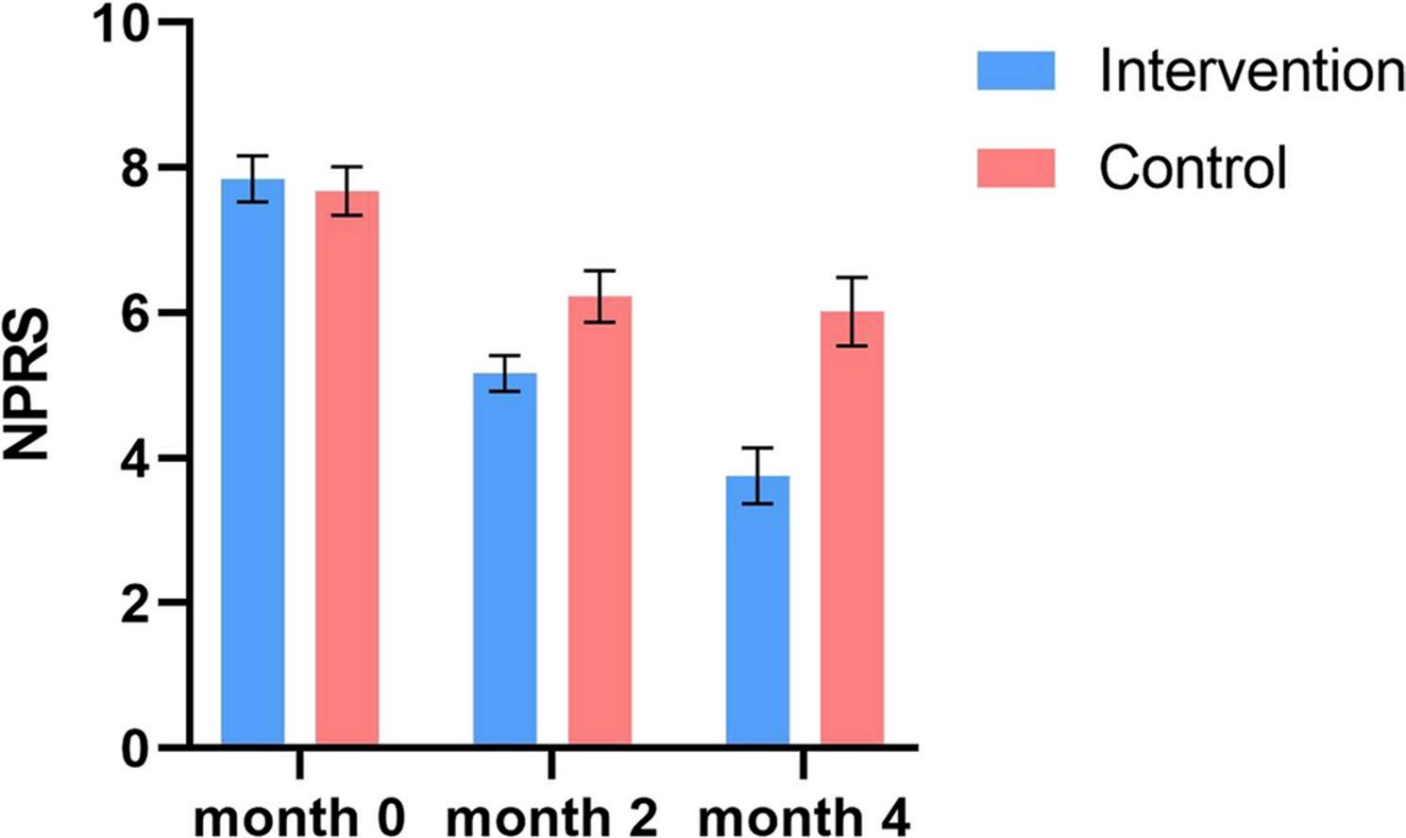
The changes in NPRS scores of patients in the intervention and control groups over the examined time period

**Fig. 3:**
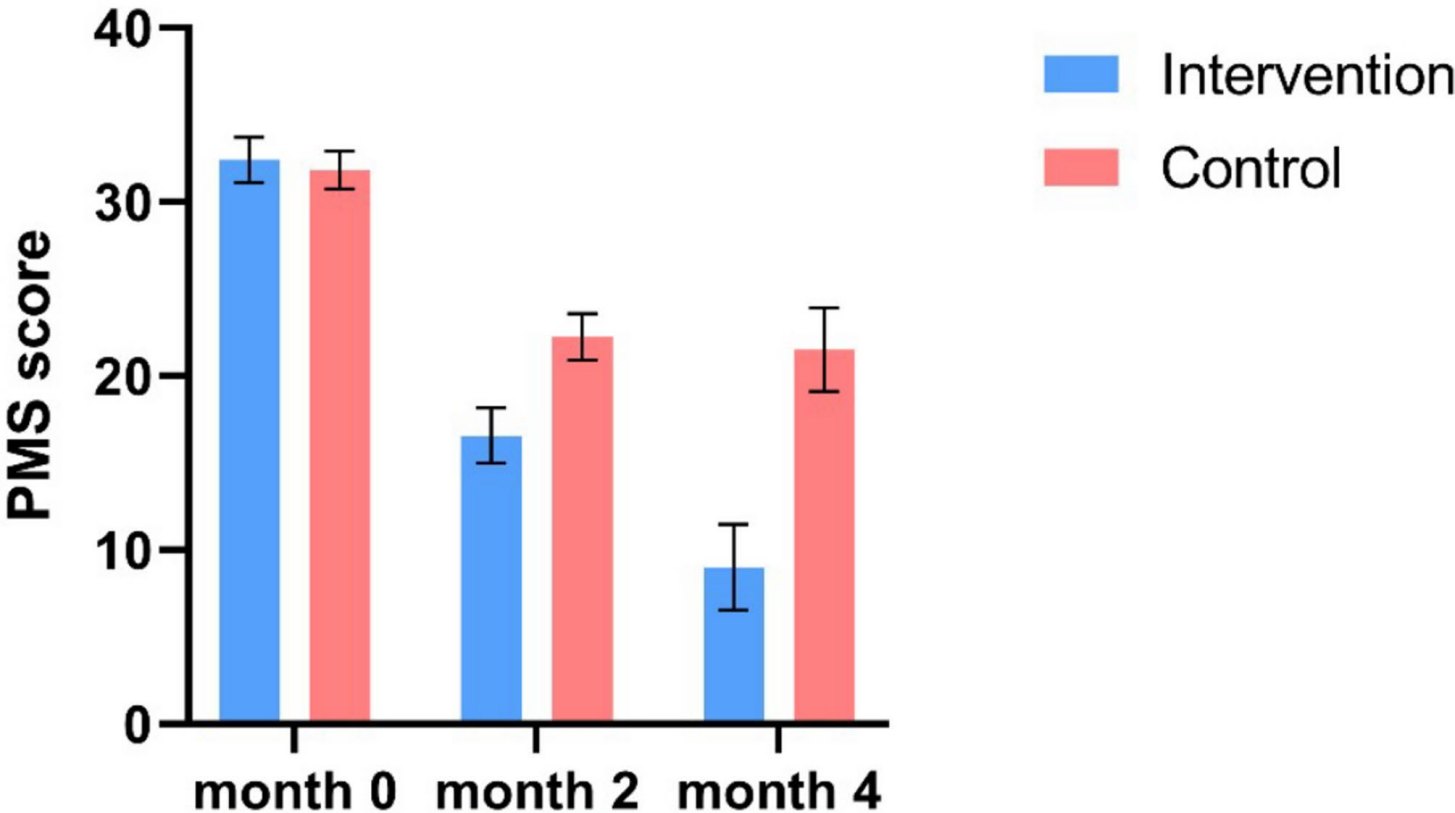
The changes in PMS scores of patients in the intervention and control groups over the examined time period

**Fig. 4:**
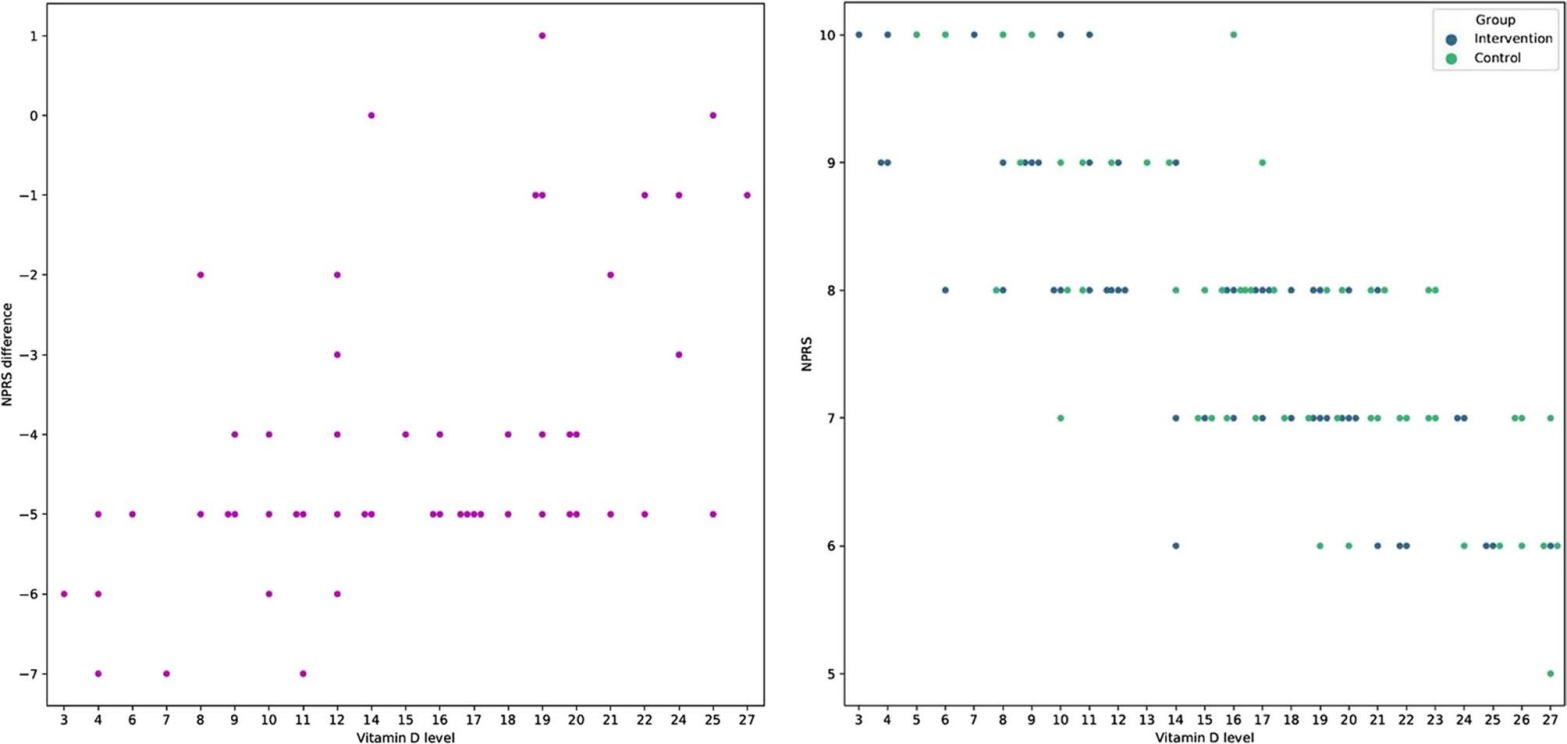
The association between vitamin D levels in the blood and patients’ NPRS scores (right plot) and the association between the initial amount of vitamin D in patients’ blood and pain decrease following a 4-month course of vitamin D and E supplementation (the left plot). The NPRS difference is calculated by subtracting the NPRS score from month one from the NPRS score from month four
